# Intestinal Chloroma

**DOI:** 10.7759/cureus.12080

**Published:** 2020-12-14

**Authors:** Erick Martín Páez Hernández, Luis Abraham Zúñiga Vázquez, Aldo Edyair Jiménez Herevia, Diego Hinojosa Ugarte, Enrique Obregón Moreno

**Affiliations:** 1 Surgery, Hospital Regional De Alta Especialidad Del Bajío, León, Guanajuato, MEX; 2 Surgical Oncology, Hospital Regional De Alta Especialidad Del Bajío, León, Guanajuato, MEX

**Keywords:** chloroma, myeloid sarcoma, granulocytic sarcoma, intestinal chloroma

## Abstract

Chloromas are an atypical cellular infiltrate of immature granulocytic cells that can occur specially in patients with acute myelogenous leukemia (AML), but can be present in nonleukemic patients. Its clinical course will be dependent on its size and location, from asymptomatic to simulating a malignant gastrointestinal neoplasia. Definitive diagnosis is made upon an immunoprofile that is similar to that present in the blasts and precursor cells of acute myeloid leukemia. Endoscopic and CT images are variable being only part of the protocol panel. Treatment is the same as to AML, but surgery and radiation must be used in order to maintain low relapse and better overall survival.

## Introduction

Chloromas, granulocytic sarcoma or myeloid sarcoma (MS) is an extramedullary solid tumor of immature granulocytic cells, known to occur commonly in patients with acute myelogenous leukemia (AML), affecting 2.5%-9.1% of cases, but it can be present in nonleukemic patients [1]. Defined by the World Health Organization (WHO) as a tumor mass consisting of myeloid blasts with or without maturation occurring at an anatomic site other than the bone marrow [2], with only 6.5% of gastrointestinal tract involvement [3].

Described more than two centuries ago by Burns, clinical and pathological diagnosis of chloroma is still highly challenging [4]. Any atypical cellular infiltrate where there was a history of myeloid neoplasia should raise the suspicion for chloroma.

We present the case of an intestinal chloroma.

## Case presentation

A 22-year-old female patient, with no significant medical history, was referred to our hospital because of growing bilateral cervical masses, odinophagy, disphony, unquantified fever, and nocturnal diaphoresis for 40 days. Three days later, petechiae were noted on her limbs, with minor transvaginal bleeding. An out of hospital bone marrow aspiration reported AML.

At physical examination, the only finding was bilateral cervical lymphadenopathy, being painful on the left side. The pulse rate was at 100 bpm, and there were no significant alterations with the rest of the vital signs. Laboratories evidenced anemia with 7 g/dL hemoglobin (range 12-15.5 g/dL), leucopenic with 0.65 x 10⁹/L of leukocytes (range 4.5-11 x 10⁹/L), profound neutropenia with 0 neutrophils (range 2,500-8,000/mm³), thrombocytopenic with platelets 22,000/µL (range 150,000-450,000/µL).

**Figure 1:**
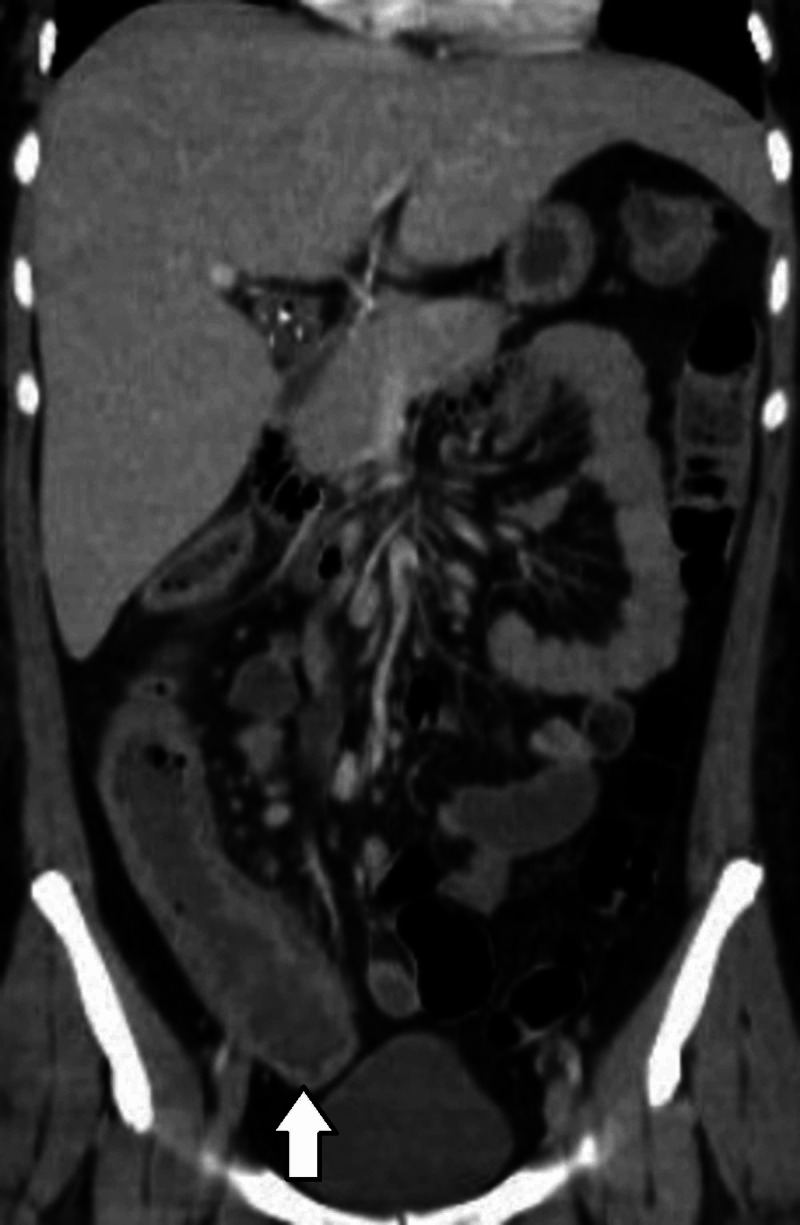
Coronal view, thickening of ascending colon.

**Figure 2:**
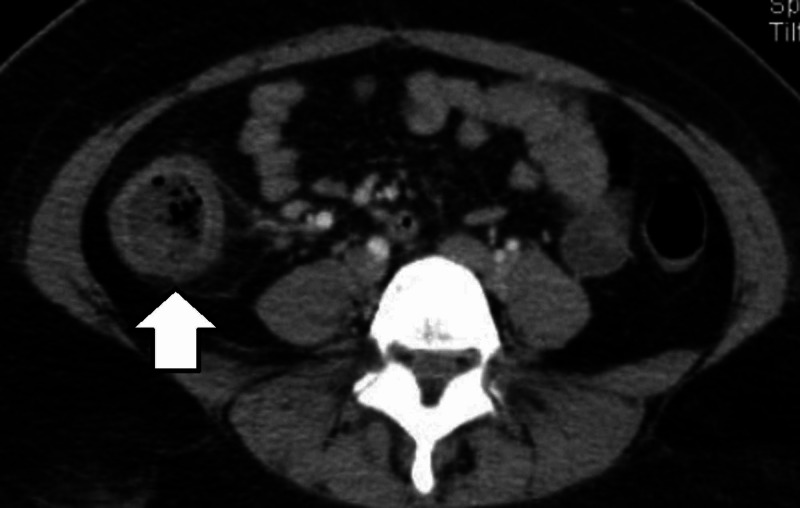
Axial view, concentric thickening in ascending colon.

Hyper-CVAD was initiated. She presented fever of 38.5°, cultures were taken as well as antibiotics initiated. She had poor evolution with persisting fever and the need of a platelet concentrate transfusion. As cultures were negative, a thoracoabdominal tomography was done with findings of inflammatory process in peritonsyllar tissue, concentric thickening of 6 mm in ascending colon and slight increase in the density of pericolic fat (Figures 1-2), neutropenic colitis that required only conservative treatment.

 

Some 10 days postadmission, she had gastrointestinal bleeding that caused persistent hypovolemic shock (with need of six blood transfusions in 24 h). Urgent surgical intervention was performed with findings of three intraluminal masses, two at 60 cm of ileocecal valve and one in the cecum; performing right hemicolectomy and ileal resection with terminal ileostomy with no complications.

She had an insidious evolution in the ICU. Some 24 h after first surgical intervention, secondary to persistent mixed hypovolemic and septic shock, a thoracoabdominal tomography is made where free abdominal fluid is evidenced. She is reintervened in the operation room, with surgical drainage of 1000 cc of citrine fluid and no other significant findings. Hours later, she died in ICU.

Surgical pathology results revealed acute colitis with microperforations, lymphatic nodes with presence of blasts, without evidence of malignancy.

**Figure 3:**
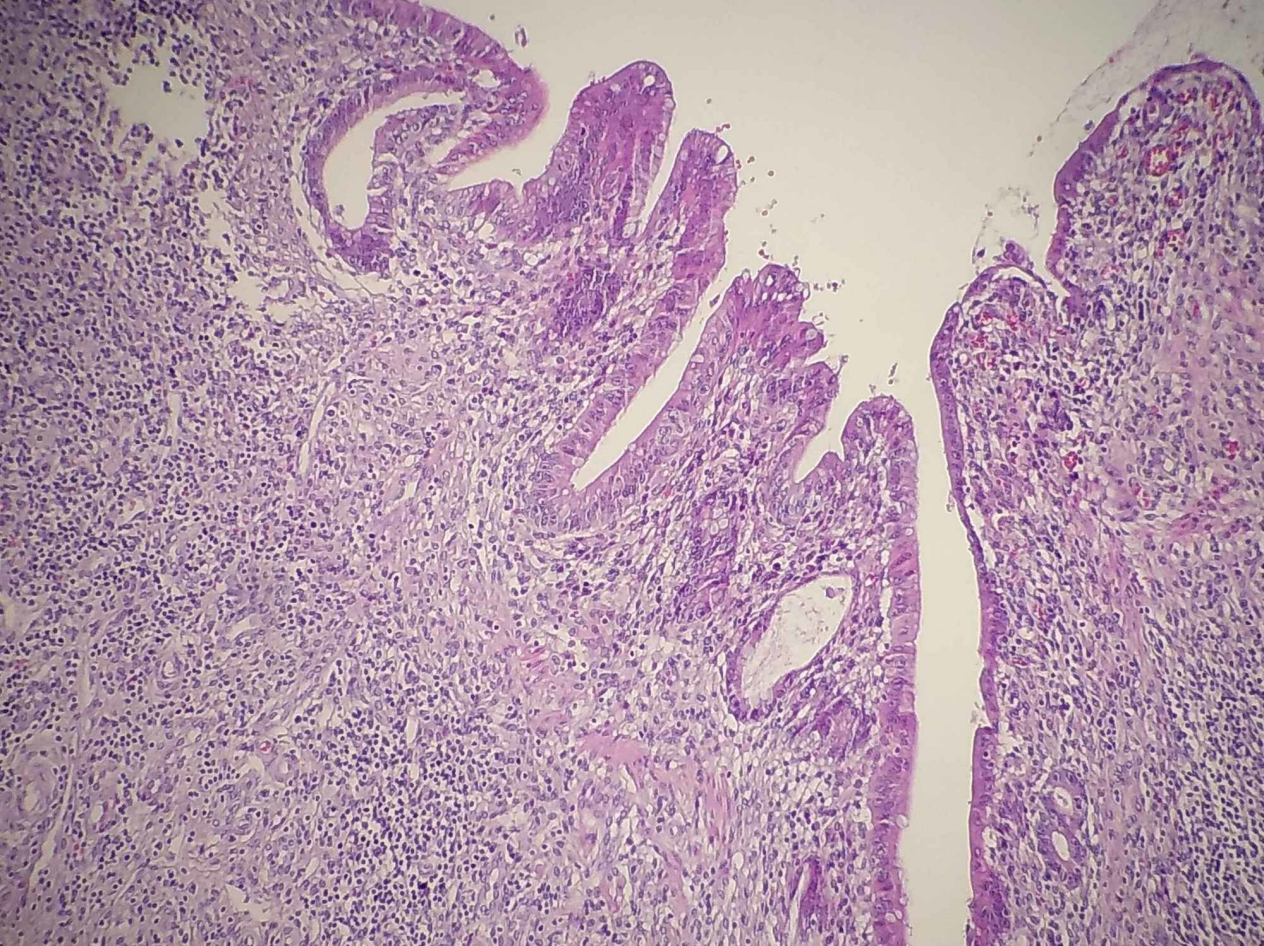
Evident infiltration of cells of wide cytoplasm and evident nucleoli in the colonic wall.

**Figure 4:**
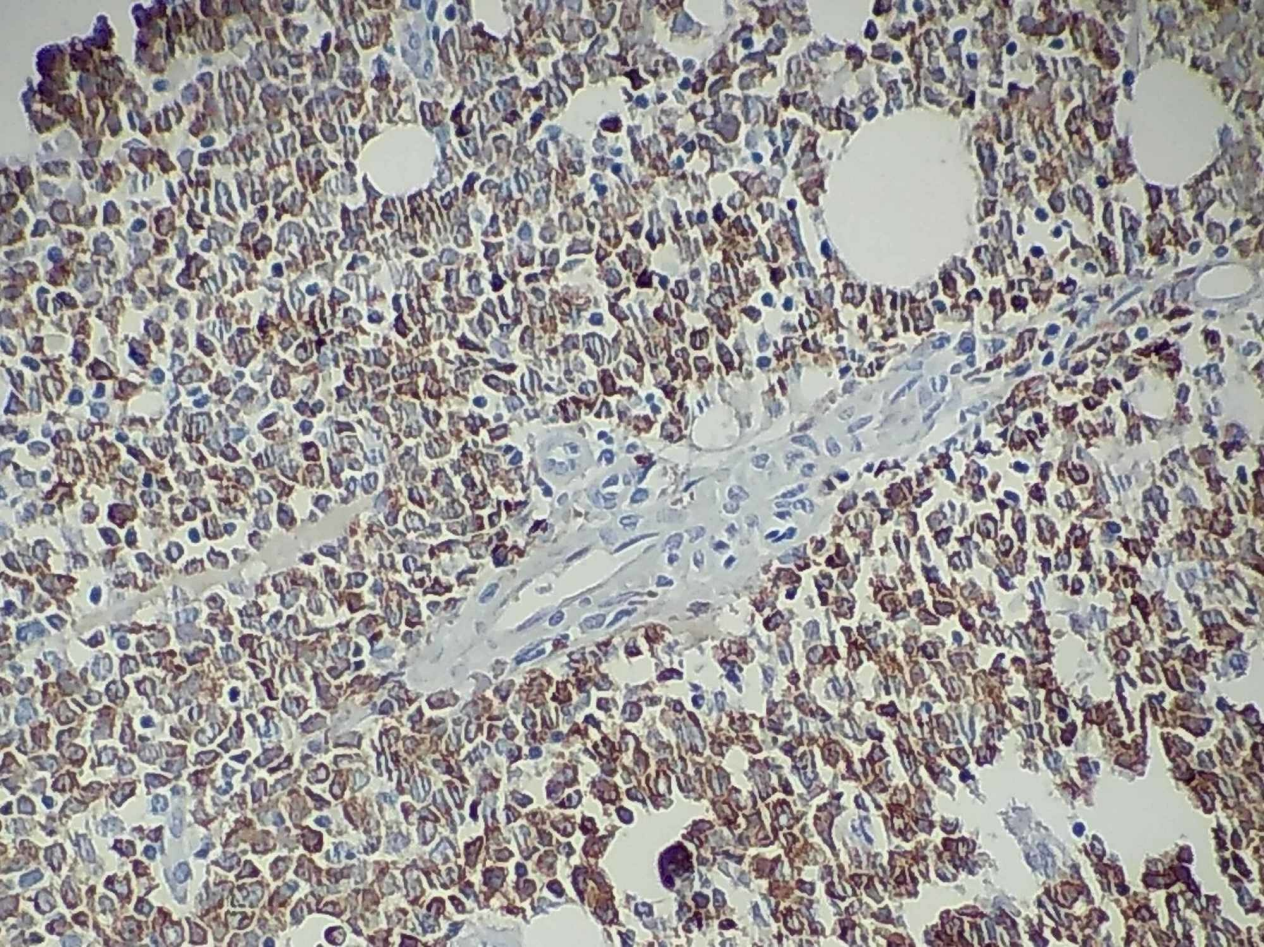
Cells intensively positive to MPO. MPO, myeloperoxidase

In histopathological sections we observe infiltration of the entire colonic wall by cells of a wide cytoplasm and evident nucleoli (Figure 3), which are intensely positive to myeloperoxidase (MPO) (Figure 4).

## Discussion

Chloromas display predominantly green color, due to the presence of MPO, as described earlier (1853) by King [5]. In 1966, Rappaport proposed the term “granulocytic sarcoma” [6], and finally, in 2002, the term myeloid sarcoma was accepted by the WHO [7]. It occurs more commonly in patients with AML (1%), less commonly in those with myelodisplastic syndrome (MDS) and myeloproliferative neoplasm. Chloromas can represent the initial manifestation of relapse in previously treated AML in remission [8]. Infrequently, extramedullary disease is the only site of involvement; the most common sites are skin, bone, soft tissue, lymph nodes. Involvement of gastrointestinal tract is rare up to 6.5% [9]. Isolated chloroma occurs in association with a normal bone marrow biopsy and blood film, in the absence of any history of myeloid neoplasia. In 90% of patients who initially had no hematological disorder, if untreated, AML develops within 10.5-11 months. Incidence of chloroma is unknown and believed to be underestimated.

Clinical presentation is varied and it is highly dependent on tumor size and location. Tumors located in gastrointestinal tract can manifest in several ways like:

· Stomach: Epigastralgy, hemorrage, perforation.

· Small intestine: Recurrent abdominal pain, subocclusive episodes, chronic diarrhoea.

· Large intestine: Simulated colorectal cancer.

Endoscopic aspects includes polipoid tumors, exofitic masses, stenosis, and ulcerations. This is why precise diagnosis is difficult [10].

The CT features of granulocytic sarcoma of the bowel are variable. Lesions appear as an intraluminal or exophytic polypoid mass, as bowel wall thickening, or as a combination of these manifestations. The enhancement patterns of these lesions are variable. Therefore, leukemic lesions of the bowel itself cannot be differentiated at CT from lymphoma or other neoplastic conditions or inflammatory bowel diseases [1].

In chloroma, several markers have been employed for identification of myeloid origin of tumor infiltration. MPO and chloroacetate immunostaining with antibodies are important for the identification of chloroma, bearing in mind that precursor cells of AML and myeloblasts in chloroma have similar antigen profile [7].

With conventional light microscopy, differential diagnosis of the pathological specimen may be difficult, particularly in the case of lymphoblastic, Burkitt’s, or diffuse large B-cell lymphoma or even a nonhematological malignancy [3].

As this leads to a completely different treatment and prognosis, immune-phenotyping is mandatory. In 92 patients, Pileri et al. demonstrated that the most commonly expressed marker was CD68/KP1 (100%), followed by MPO (83.6%), CD117 (80.4%), CD99 (54.4%), CD68/PG-M1 (51.0%), and CD (43.4%) [11].

Antic et al. suggested that the minimum panel of immunohistochemical markers should include MPO, CD68, CD43, lysozyme, CD33, CD34, and CD117 in order to establish a definitive diagnosis [7].

There are no consensus guidelines or protocol regarding chloroma treatment. This will depend on several factors including: location of disease, whether it is a relapse or an initial diagnosis, age of patient, and performance status. Current treatment regimen in patients with chloroma or isolated chloroma is conventional AML chemotherapeutic protocols with or without radiotherapy. Surgery can be considered before systemic treatment when rapid debulking, symptomatic relief is needed or to confirm diagnosis. Hematopoietic stem cell transplantation (HSCT) should also be considered in relapsed or refractory patients or even as a consolidation treatment [12].

## Conclusions

Although relatively uncommon, chloroma should be suspected in every patient with myeloid neoplasia history with added symptoms. Even though the clinical manifestations are variable, image should always be part of the diagnostic arsenal in order to establish therapeutic regimen that must include surgical, radiation, and chemotherapy. Treatment guidelines based on prospective studies of this rare manifestation are required to determine the best combination of actual therapies, as early detection and treatment will lead to a better overall patient's survival and quality of life.
